# A novel nanotechnology-based strategy using CNT-Fe_3_O_4_ for early and accurate detection of EGFR T790M mutation in NSCLC

**DOI:** 10.1016/j.jgeb.2026.100697

**Published:** 2026-04-22

**Authors:** Saja S. Falih, Ahmed Mahdi Rheima, Fatin Fadhel Al-Kazazz, Zaid Nsaif Abbas

**Affiliations:** a Department of Chemistry, College of Science, Mustansiriyah University, Baghdad, Iraq; b College of Biotechnology, Al-Nahrain University, Baghdad, Iraq

**Keywords:** Carbon nanotubes (CNTs), Fe_3_O_4_ nanoparticles, Nanocomposite biosensor, EGFR T790M mutation, Non-small cell lung cancer (NSCLC), EDC/NHS coupling, Fluorescence hybridization assay

## Abstract

A magnetically responsive carbon nanotube–iron oxide (CNT–Fe_3_O_4_) nanocomposite was synthesized and surface-functionalized with citric acid to introduce carboxyl groups suitable for stable bio conjugation. A mutation-specific amine-modified oligonucleotide probe targeting the EGFR T790M resistance mutation was covalently immobilized onto the activated nanocomposite using EDC/NHS coupling, forming a selective molecular biosensing platform. DNA extracted from FFPE tumor tissues of NSCLC patients was evaluated through a fluorescence-based hybridization assay integrated with magnetic separation to reduce nonspecific adsorption. T790M-positive samples showed clearly higher measured DNA concentration values after hybridization (5.9–7.4 ng/µL; mean = 6.7 ± 0.6 ng/µL), whereas negative and control samples remained low (1.7–2.3 ng/µL), confirming the analytical specificity and reproducibility of the developed system.This platform offers a rapid, cost-effective, and portable approach for mutation detection without requiring complex instrumentation. The CNT–Fe_3_O_4_–probe nanoplatform demonstrates promising potential for future translation into liquid biopsy applications and personalized therapeutic monitoring in NSCLC.

## 1 Introduction

NSCLC is the most common type of lung cancer, making up to 85% of all lung cancers in the world and is still one of the leading causes for ages-standardized cancer death with over 1.8 million deaths per year.^1^, ^2^, ^3^ At present, EGFR-targeting drugs have brought good prospects for patients, however, resistance would frequently occurs after a period of time (acquired resistance), with the most frequent mechanism being T790M mutation, occurring in about 50–60% non-small cell lung cancer (NSCLC) patients who are treated with first and second generation agents that inhibit EGFR signaling^4^, ^5^, ^6^. his mutation is due to a T-to-M substitution at position 790 and enhances ATP-binding affinity while reducing drug-effect, hence requiring therapy change from first-line TKIs to third-generation drugs like Osimertinib that has shown better clinical efficacy in pivotal studies.^7^, ^8^

Thus, the detection of T790M mutation is a pivotal prerequisite to make therapeutic decisions. Current diagnostic methodologies, such as liquid biopsy based on circulating free DNA (cfDNA) analysis through digital droplet PCR (ddPCR), next-generation sequencing (NGS), or commercial assays serve as valuable non-invasive options, but all have limited sensitivity, and still fail to detect mutation when the mutant allele fraction of plasma is below a certain level. Published sensitivities are approximately 60–65%, which is slightly lower than ddPCR (≈88%) and NGS (>90%) based assays.^9^, ^10^, ^11^, ^12^ These limitations lead to false-negative findings and emphasize a pressing requirement for new analytical systems with enhanced sensitivity and single nucleotide resolution.

Nanotechnology offers encouraging prospects for overcoming these challenges. Magnetic Fe_3_O_4_ nanoparticles can facilitate the quick enrichment and separation of target molecules in an external magnetic field, while carbon nanotubes (CNTs) as good conductive materials with large surfaces help improve the stability of probes and signal strength. The incorporation of these nanomaterials leads to a hybrid platform that involves magnetic enrichment based on the robust molecular immobilization of the target molecules.^13^, ^14^

Based on this idea, we engineered a nano-probe that is complementary only to the EGFR T790M. It binds specifically to the mutant allele in the sample, following addition, thus allowing clear delineation from the wild-type sequence even at single base resolution. The prepared complexes are magnetically enriched by Fe_3_O_4_ NPs, stabilized with CNTs, and finally quantified via fluorometric detection. The fluorometric readout is based on the production or extinction of fluorescence signals during hybridization, and enables sensitive detection and quantification of T790M point mutations even at low concentrations.

## 2 Experimental

### 2.1 DNA extraction from FFPE lung cancer samples

Formalin-fixed paraffin-embedded (FFPE) tissue sections from confirmed non-small cell lung cancer (NSCLC) patients were used as the DNA source. DNA extraction was performed using the AmoyDx® FFPE DNA Kit (Amoy Diagnostics, China) according to the manufacturer’s instructions. Briefly, 5–10 µm thick FFPE sections were deparaffinized using xylene, followed by sequential ethanol washes for rehydration. The tissue pellets were then lysed in a digestion buffer containing Proteinase K and incubated at 56 °C until complete tissue dissolution. DNA was subsequently purified through silica-based spin columns, eluted in nuclease-free water, and quantified using a Quantus™ Fluorometer (Promega, USA), a pre-calibrated fluorometric system that provides direct concentration readouts based on the manufacturer’s assay chemistry. Because FFPE-derived DNA is often fragmented and present in limited amounts, fluorometric quantification was used as the primary method for DNA assessment, as it is more suitable for degraded, low-input samples than conventional spectrophotometric purity measurements such as A_260_/A_280_. DNA integrity was assessed by agarose gel electrophoresis.

### 2.2 Detection of EGFR T790M mutation by super-ARMS® PCR

To identify the presence of the EGFR T790M mutation, all extracted DNA samples were analyzed using the Super-ARMS® EGFR Mutation Detection Kit (Amoy Diagnostics, China). The assay utilizes a proprietary amplification-refractory mutation system designed for high-specificity detection of single-nucleotide variants. PCR amplification was carried out on a QuantStudio 5 Real-Time PCR System (Applied Biosystems, USA) using the following thermal profile: initial denaturation at 95 °C for 5 min, followed by 40 cycles of 95 °C for 15 s and 60 °C for 60 s. Fluorescence signals were analyzed according to the manufacturer’s threshold criteria. Samples with amplification in the mutant channel (ΔCt ≤ 5) were considered T790M-positive, while samples showing amplification only in the wild-type channel were classified as T790M-negative.

### 2.3 Preparation of positive and negative DNA templates

DNA extracted from T790M-positive patients, previously identified by Super-ARMS® PCR, was used as the positive template, whereas DNA from T790M-negative (wild-type) patients was used as the negative template in the nanoparticle–probe hybridization assay. To generate defined DNA templates for subsequent nanoparticle-based detection, specific fragments of the EGFR gene encompassing the T790M locus were amplified using the following primer set:

Forward primer: 5′-AGGAAGCCTACGTGATG-3′.

Reverse primer: 5′-CCAGTTGAGCAGGTACT-3′.

PCR amplification was carried out in a total reaction volume of 25 µL containing 12.5 µL of 2 × PCR Master Mix, 1 µL of each primer (10 µM), 2 µL of DNA template, and nuclease-free water to the final volume. The thermal cycling program consisted of an initial denaturation step at 95 °C for 5 min, followed by 30 cycles of 94 °C for 30 s and 60 °C for 1 min 30 s, with a final hold at 10 °C. The amplified product was approximately 170 bp, which is appropriate for FFPE-derived DNA because of its fragmented nature.

Amplification success and product specificity were verified by 2% agarose gel electrophoresis, where a distinct band of the expected size was observed. The PCR products were then purified using the Wizard® SV Gel and PCR Clean-Up System to remove residual primers, nucleotides, and enzymes. The purified amplicons were subsequently used as standardized positive and negative DNA templates in the nanoparticle–probe hybridization assay. Each template was adjusted to a final concentration of 20 ng/µL in TE buffer (10 mM Tris-HCl, 1 mM EDTA, pH 8.0) and stored at − 20 °C until use. The mutation status of the templates was assigned based on prior Super-ARMS® PCR results.

### 2.4 Synthesis of CNT–Fe_3_O_4_ nanocomposite

#### 2.4.1 Sustainable bio-mass synthesis of carbon nanotubes (CNTs)

**Fig. 1:**
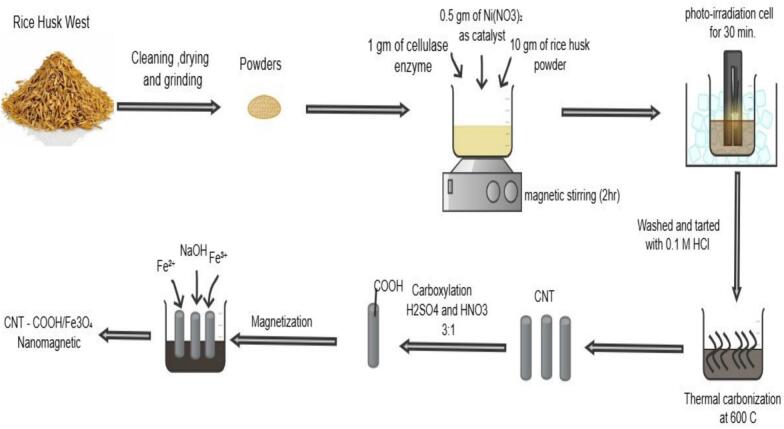
Graphitic steps of the synthesis of carbon nanotubes (CNTs).

Carbon nanotubes (CNTs) were synthesized from rice husk biomass using a green and sustainable approach (Fig. 1). The rice husk was thoroughly washed with deionized water to remove contaminants and oven-dried at 100–110 °C for 3 h to eliminate residual moisture. The dried husk was then ground into a fine powder to increase the surface area for enzymatic hydrolysis. Subsequently, 10 g of the powdered rice husk was suspended in 100 mL of deionized water, and 1 g of cellulase enzyme was added under continuous stirring for 2 h. This enzymatic treatment facilitated the breakdown of cellulose fibers and increased the amount of carbonaceous material. Next, 0.5 g of Ni(NO_3_)_2_ was added as a catalyst precursor, and the mixture was stirred for an additional 30 min. The resulting suspension was then transferred into a UV irradiation chamber equipped with a 125 W mercury lamp (365 nm) and irradiated for 30 min under continuous cooling using an ice bath to avoid overheating during the irradiation process. This irradiation step promoted the formation of carbonaceous nanostructures and facilitated CNT nucleation. The resulting precipitate was washed thoroughly several times with deionized water and treated with 0.1 M HCl to remove residual metal ions and impurities. Finally, the material was thermally carbonized at 600 °C under atmospheric conditions, using a heating rate of 5 °C/min and a dwell time of 3 h, resulting in the formation of CNTs with the aid of the residual nickel catalyst, which promoted nanotube growth. This method integrates enzymatic hydrolysis, catalytic activation, UV irradiation, and controlled thermal treatment with relatively low energy and water consumption, providing an economical and environmentally friendly route for producing CNTs from biomass for applications in nanotechnology, catalysis, and environmental remediation.

#### 2.4.2 Carboxylation of carbon nanotubes (CNTs) via acid oxidation

To introduce carboxyl (–COOH) functional groups onto the surface of carbon nanotubes (CNTs), an acid oxidation treatment was performed using a mixture of strong acids. Initially, 0.5 g of CNTs was dispersed in 100 mL of a 3:1 mixture of concentrated sulfuric acid (H_2_SO_4_) and nitric acid (HNO_3_). The mixture was then subjected to ultrasonication for 30 min to enhance acid penetration and oxidation efficiency. Subsequently, the mixture was heated to 70-80 °C under continuous stirring for 4 h to ensure effective oxidation. During this process, the strong acids generated oxidative species that selectively cleaved C–C bonds at defect sites on the CNT walls, leading to the formation of carboxyl (–COOH) and hydroxyl (–OH) functional groups on the surface. After the reaction was completed, the mixture was cooled to room temperature and then carefully diluted with deionised water. The precipitate was separated using a centrifuge, followed by multiple washing cycles via decantation with deionized water until the filtrate reached a neutral pH (∼7). Finally, the sample was dried at 80 °C for 6 h, yielding carboxyl-functionalized CNTs (CNT-COOH).

#### 2.4.3 Magnetization of carbon nanotubes (CNTs) with Fe_3_O_4_ nanoparticles

**Fig. 2:**
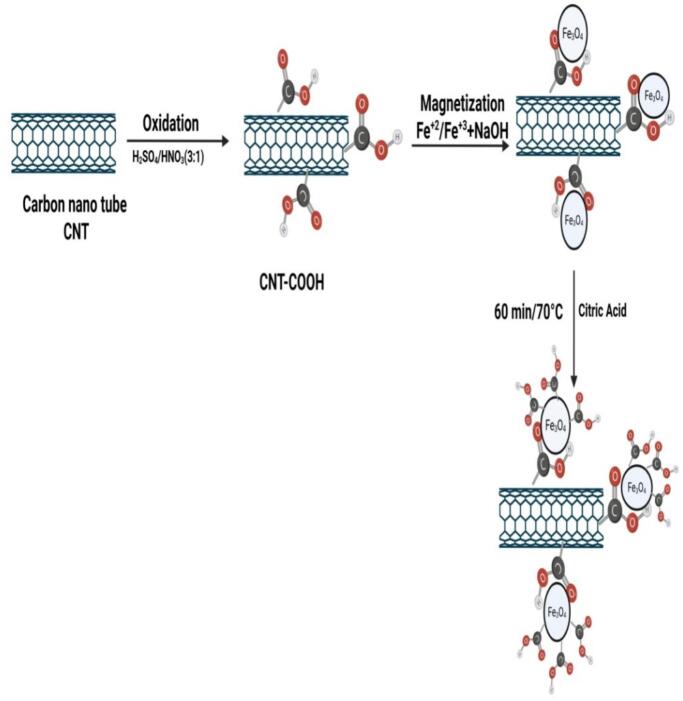
Schematic representation of CNT–Fe_3_O_4_ synthesis and surface modification for probe conjugation.

To impart magnetic properties to carbon nanotubes (CNTs), Fe_3_O_4_ nanoparticles were deposited onto their surface using the co-precipitation method (Fig. 1). Initially, 0.5 g of carboxyl-functionalized CNTs was dispersed in 100 mL of deionized water and subjected to ultrasonication for 30 min to ensure uniform dispersion. Accordingly, 2 g of ammonium ferrous sulfate ((NH_4_)_2_SO_4_·FeSO_4_·6H_2_O) was dissolved in 25 mL of deionized water, and 4.8 g of ammonium ferric sulfate (NH_4_Fe(SO_4_)_2_·12H_2_O) was dissolved in 25 mL of deionized water. The two solutions were then mixed under continuous stirring at room temperature and gradually added to the CNT suspension while stirring for 30 min, facilitating interaction between CNTs and Fe^2+^/Fe^3+^ ions as a 1:2. Subsequently, 1 M sodium hydroxide (NaOH) solution was added drop wise until the pH reached ∼10, leading to the co-precipitation of Fe_3_O_4_ nanoparticles onto the CNT surface. The reaction was maintained under continuous stirring at room temperature for 1 h to ensure the complete formation and efficient attachment of Fe_3_O_4_ onto the CNTs. Following precipitation, the CNT–Fe_3_O_4_ composite was separated by centrifugation at 6000 rpm for 5 min, and this step was repeated three times. Magnetic separation was not employed at this stage; the composite was isolated by centrifugation only. The collected product was then washed several times with deionized water and ethanol to remove unreacted precursors and impurities. Finally, the material was dried at 80 °C for 6 h, yielding a magnetically responsive CNT–Fe_3_O_4_ composite. This hybrid material combines the high electrical conductivity and large surface area of CNTs with the superparamagnetic behaviour of Fe_3_O_4_, making it suitable for various applications, including magnetic separation, drug delivery, wastewater treatment, and nanocomposites. The obtained material was then functionalized with citric acid in an aqueous medium at 70 °C for 60 min to generate additional free carboxyl groups on the surface, which were later utilized for covalent probe conjugation through amide bond formation. After functionalization, the product was washed several times with deionized water until the pH of the washings reached 7 to remove excess citric acid and unreacted byproducts. The purified material was then dried before further use.(Fig. 2).

### 2.5 Activation of carboxyl groups on nanoparticles

To activate the carboxyl groups on the surface of the nanoparticles, freshly prepared solutions of N-hydroxysuccinimide (NHS) and 1-ethyl-3-(3-dimethylaminopropyl)carbodiimide (EDC) were used. Specifically, 0.5 mg of NHS was dissolved in 5 mL of deionized water to obtain a concentration of approximately 0.1 mg/mL (0.87 mM), and 0.35 mg of EDC was dissolved in 4.65 mL of deionized water to obtain a concentration of approximately 0.075 mg/mL (0.39 mM). From each solution, 50 µL was used for the activation step. The activation reaction was performed in an aqueous medium using deionized water.

### 2.6 Preparation and activation of nanoparticle suspension

A total of 100 µL of nanoparticles was mixed with 800 µL of TE buffer. The prepared NHS and EDC solutions were then added to the nanoparticle suspension. The mixture was stirred gently for 15 min at room temperature to allow activation of the carboxyl groups on the nanoparticle surfaces. Successful activation was inferred indirectly from the subsequent covalent probe conjugation and the measurable ssDNA-associated signal obtained after the conjugation and washing steps.

### 2.7 Conjugation of probes to activated nanoparticles

**Fig. 3:**
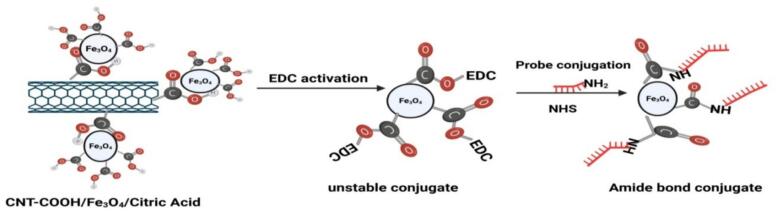
Schematic illustration of the activation and probe conjugation process of CNT–COOH/Fe_3_O_4_/citric acid nanocomposites. The carboxyl groups on the nanoparticle surface were activated by EDC/NHS chemistry to form an unstable intermediate, which subsequently reacted with the amino-modified probe (–NH_2_ C6) to form a stable amide bond conjugate.

The probe specific for EGFR T790M mutation detection was synthesized by Macrogen (Korea) with the following sequence and modification features: T790M-P probe, 5′-FAM-ATGAGCTGCATGATGAG-NH_2_ C6-3′. This probe was designed to specifically target the EGFR T790M mutant sequence, corresponding to the c.2369C>T (p.Thr790Met) substitution in exon 20 of the EGFR gene. Following activation, 10 µL of the activated nanoparticle suspension was mixed with 10 µL of the probe solution in a single microtube. The mixture was gently vortexed and incubated overnight at room temperature to promote covalent attachment of the amino-modified probe to the carboxyl-activated nanoparticle surface through stable amide bond formation. The –NH_2_ C6 modification at the 3′ end facilitated covalent linkage with the activated nanoparticles, while the 5′-FAM fluorophore served as the fluorescent reporter in subsequent hybridization and detection assays. Probe conjugation was confirmed using a fluorometric ssDNA dye-based assay after the conjugation and washing steps. The initial probe stock concentration before conjugation was 140 ng/µL. After conjugation and repeated washing to remove unbound probe, the probe–nanoparticle preparation showed a measurable ssDNA-associated concentration of approximately 8.9 ng/µL, indicating successful probe immobilization on the nanoparticle surface. The functionality of the conjugated probe was further supported by the subsequent hybridization assay (Fig. 3).

### 2.8 Washing and purification of conjugated nanoparticles

Once the conjugation was complete, the NP was isolated by magnetic separation. Tubes were put on a magnetic rack to obtain the nanoparticles and the supernatant consisting of unbound probes was decanted. The nanoparticles were washed twice with TE buffer to eliminate free materials.

### 2.9 Hybridization of probe-nano complex with template DNA

For hybridization, 5 µL of the nanoparticle–probe complex was mixed with 5 µL of template DNA and 10 µL of distilled water. The mixture was then subjected to thermal treatment in a thermal cycler under the following conditions: denaturation at 95 °C for 5 min, annealing at 60 °C for 3 min, and cooling at 4 °C for 2 min.

### 2.10 Final washing and quantification

After thermal treatment, the nanoparticle complexes were washed twice with TE buffer using magnetic separation to remove unbound DNA. The resulting nanoparticle–probe–template complex was then resuspended in 10 µL of TE buffer. For probe–template hybridization quantification, 1 µL of the final product was mixed with 200 µL of dsDNA-binding dye, and the sample was measured using a fluorometer. The reported values were recorded as fluorescence-derived DNA concentration outputs.

## 3 Results and discussion

### 3.1 Field emission scanning electron microscope of CNT-Fe_3_O_4_

**Fig. 4:**
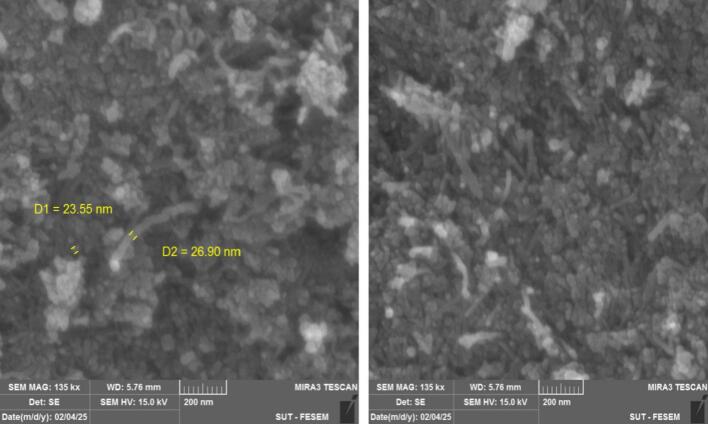
FE-SEM images of CNT-Fe_3_O_4_ Nanostructures.

Morphology of the CNT–Fe_3_O_4_ hybrid was characterized by field-emission scanning electron microscopy (FE-SEM). Prior to imaging, the samples were gold-coated to improve conductivity, and the SEM analysis was performed at an accelerating voltage of 15 kV. Representative FE-SEM images with appropriate scale bars are shown in Fig. 4.The SEM images directly evidence the successful construction of a CNT–Fe_3_O_4_ hybrid nanostructure, in agreement with prior reports on Fe_3_O_4_-decorated CNT frameworks.^15^, ^16^ The CNTs form a labyrinth-like, highly entangled network that provides a large accessible surface area for anchoring Fe_3_O_4_ nanoparticles.^15^, ^17^ FE-SEM images show Fe_3_O_4_ nanoparticles attached to CNT sidewalls, with well-dispersed deposits in many regions and point agglomeration elsewhere behavior commonly observed for in-situ or co-precipitated Fe_3_O_4_ on CNTs and influenced by surface functionalization.^18^, ^19^, ^20^ The particle size of Fe_3_O_4_ lies between 20–32 nm, confirming the nanoscale feature and aligning with typical magnetite sizes reported for CNT–Fe_3_O_4_ nanocomposites and wet-chemical routes.^18^, ^21^ The interaction between Fe_3_O_4_ and CNTs appears sufficiently strong to create nucleation/anchoring sites on the nanotube walls when oxygenated functionality is present (e.g., –COOH or polydopamine-derived catechols), consistent with literature on carboxylated CNTs and PDA-assisted anchoring.^22^ The observed heterogeneity of Fe_3_O_4_ distribution (coexistence of dispersed nanoparticles and local clusters) is consistent with differences in reaction conditions and functionalization strategies.^22^ Despite local agglomeration, the CNT scaffold maintains flexibility while Fe_3_O_4_ loading enhances the composite’s magnetic responsiveness, as reported for magnetic CNTs.^15^ Overall, FE-SEM analysis indicates that Fe_3_O_4_ nanoparticles are effectively hybridized with CNTs to form a nanostructured composite, and the size range and interfacial architecture suggest suitability for magnetic nanocomposites, catalysis, and environmental/biomedical platforms.^15^, ^23^

### 3.2 Transmission electron microscopy (TEM) analysis of CNT-Fe_3_O_4_ nanocomposites

**Fig. 5:**
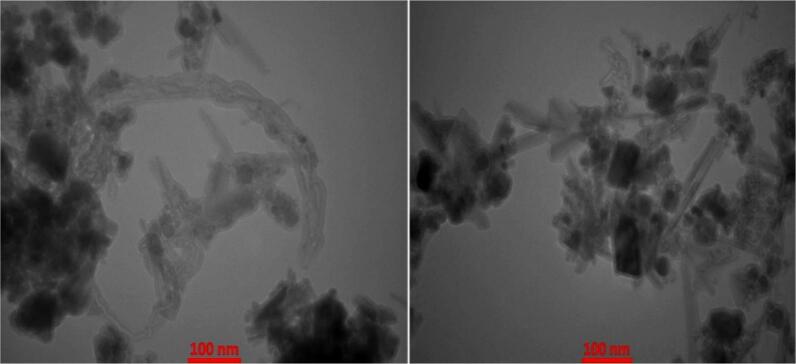
TEM Analysis of CNT-Fe_3_O_4_ Nanostructures.

A transmission electron microscope (TEM) was used to obtain detailed information about the size, shape, and structure of the synthesized CNT-Fe_3_O_4_ nanostructures. The TEM size images in Fig. 5 indicate the unique morphology of well-defined tubular structures of the CNT-Fe_3_O_4_ nanostructures. A little bit of agglomeration can be noted due to the interaction between Fe_3_O_4_ nanoparticles and CNTs, and the synthesis method (commonly observed in Fe_3_O_4_–CNT systems; strategies to minimize it are reported in.^24^ The mean particle size of the Fe_3_O_4_ nanoparticles obtained from the TEM images selected at random was an average of 23 nm. The results are strongly supported by X-ray diffraction (XRD) profile that has been analysed using Debye–Scherrer equation, which reveals that Fe_3_O_4_ nanoparticles are well integrated on the surface of CNT without altering into the nano state (XRD/Scherrer assignment of Fe_3_O_4_ on CNTs.^25^, ^26^

### 3.3 X-ray diffraction of CNTs \ Fe_3_O_4_ nanoparticles

**Fig. 6:**
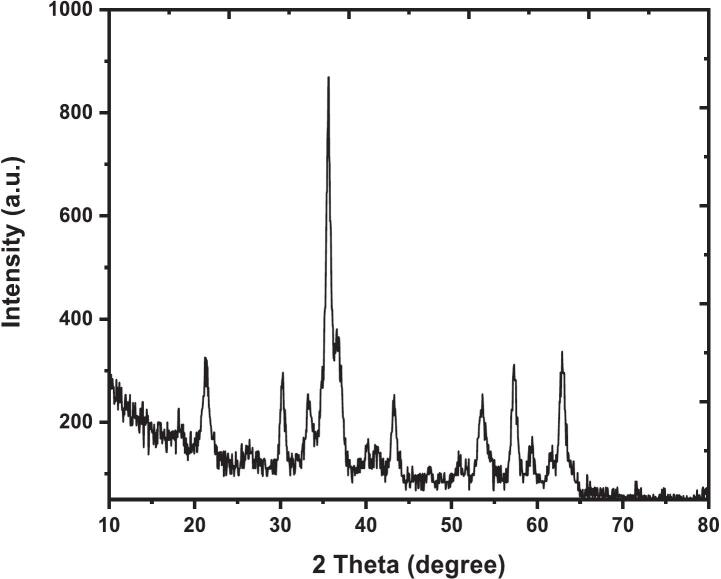
XRD Pattern of CNT \Fe_3_O_4_ Nano-magnetic.

X-ray diffraction was performed to confirm the crystalline nature of the CNTs hybrid with Fe_3_O_4_ nanoparticles. Fig. 6 shows the XRD pattern of the CNT hybrid with Fe_3_O_4_ nanoparticles. The contribution of CNTs was assessed by comparison with a separate XRD pattern obtained for pristine CNTs, which showed characteristic graphitic reflections at 2θ = 26.06° and 43.34°, corresponding to the (002) and (100) planes, respectively. As shown in the spectrum, the diffraction peaks are attributed to the graphitic carbon from CNTs and magnetite from Fe_3_O_4_, respectively, in agreement with standard profiles of CNTs and inverse-spinel Fe_3_O_4_.^27^, ^28^ The strongest and prominent diffraction peak was observed at 2θ = 43.27, which corresponds to the (100) plane of pure graphitic carbon as reported in JCPDS card #96-101-1061 for CNTs, indicating that CNTs retained the crystalline structure after the hybridization with Fe_3_O_4_^27^, ^29^; we note that Fe_3_O_4_ also shows a (400) reflection near 43.1° (JCPDS85-1436), so partial overlap is commonly discussed in the literature.^30^ In addition, the incorporation of Fe_3_O_4_ into the hybrid was confirmed by the distinctiveness of observed peaks that match the inverse spinel structure of magnetite; the diffraction peak at 2θ ≈ 30.18 with an interplanar spacing near ∼2.96 Å corresponds to the (220) plane of Fe_3_O_4_.^30^, ^31^ The most intense peak at 35.61 corresponds to the (311) plane, a crystal-plane fingerprint of Fe_3_O_4_, and another intense peak at 36.89 matches the (222) plane, in line with standard data. Other characteristic peaks at 53.47, 57.28, and 62.98 were observed and matched the (422), (511), and (440) planes of Fe_3_O_4_, respectively, further confirming the presence of Fe_3_O_4_.^30^, ^31^ The Debye–Scherrer formula was used to estimate the crystallite size of Fe_3_O_4_ from the (311) reflection at 35.61° in the XRD pattern of the synthesized CNT–Fe_3_O_4_ composite. No separate instrumental broadening correction was applied in the present calculation; therefore, the reported crystallite size (∼22.78 nm) should be considered an approximate estimate.^32^, ^33^ The broadening of peaks shows the synthesized CNT–Fe_3_O_4_ hybrid is of a nanoscale size due to small particle sizes and effective dispersion of Fe_3_O_4_ on the CNT surface, as commonly interpreted via size/strain models.^32^, ^33^ From the XRD results, it is observed that CNT–Fe_3_O_4_ hybrid nanocomposite was formed successfully. These structural findings are consistent with recent studies on iron oxide-based hybrid nanocomposites, where complementary characterization techniques such as XRD, SEM, FTIR, and UV–Vis were collectively employed to confirm crystallinity, nanoscale morphology, and successful composite formation. For instance, ZnO@Fe_3_O_4_, Fe_3_O_4_/NiO, and Ag@Fe_3_O_4_ nanocomposites have been reported to exhibit well-defined crystalline structures and particle sizes within the nanometer range, confirming the reproducible formation of stable multicomponent nanomaterials.^34^, ^35^, ^36^ In agreement with these reports, the FE-SEM, TEM, and XRD analyses obtained in the present work confirm the successful integration of Fe_3_O_4_ nanoparticles onto the CNT framework, indicating the formation of a stable CNT–Fe_3_O_4_ hybrid nanocomposite suitable for subsequent probe conjugation and mutation detection.

The sharp and well-defined peaks also reveal a good crystallinity that makes it suitable for several applications, such as magnetic nanocomposites, catalysis, nanomedicine, water treatment, and energy storage, in line with recent reports on Fe_3_O_4_-based functional composites.^37^, ^38^ Carboxylated CNTs provide negatively charged sites that attract Fe^2+^/Fe^3+^ ions, favouring heterogeneous nucleation of Fe_3_O_4_ on the CNT walls. The nanometric crystallite size and the absence of secondary iron oxide phases confirm well-controlled co‑precipitation, enhancing magnetic responsiveness and ion‑transport efficiency.

### 3.4 Fluorescence-based detection of EGFR T790M using CNT–Fe_3_O_4_–probe nanoplatform

The quantitative fluorescence outcomes obtained from the nanoparticle–probe hybridization assay are presented in Table 1.

**Table 1:** : Quantification Results of Probe-Nanoparticle Hybridization.

Sample	Description	DNA Concentration (ng/µL)	Interpretation
Positive 1 (+)	Nanoparticle–probe + Template DNA (Patient A)	6.8	Successful hybridization indicating the presence of T790M mutation
Negative 1 (–)	Nanoparticle–probe + wild-type template DNA (Patient A)	1.7	Low concentration value confirming absence of mutation
Control (H_2_O)	Nanoparticle–probe + distilled water	2.3	Minimal non-specific binding (system background)
Positive 2 (+)	Nanoparticle–probe + Template DNA (Patient B)	7.4	High concentration value confirming the presence of the T790M mutation
Negative 2 (–)	Nanoparticle–probe + wild-type template DNA (Patient B)	2.1	Low concentration value indicating absence of mutation and high probe specificity
Positive 3 (+)	Nanoparticle–probe + Template DNA (Patient C)	5.9	Elevated concentration value indicating successful detection of the mutant allele
Negative 3 (–)	Nanoparticle–probe + wild-type template DNA (Patient C)	1.9	Low concentration value confirming absence of mutation

The conjugation and hybridization experiments demonstrated the ability of the CNT–Fe_3_O_4_–probe system to discriminate between EGFR T790M-positive and wild-type samples. Among the three T790M-positive DNA templates, fluorometer-derived DNA concentration values were consistently observed, ranging from 5.9 to 7.4 ng/µL (mean = 6.7 ± 0.6 ng/µL). These results support successful hybridization between the nanoparticle–probe complex and the mutant DNA sequences under the applied experimental conditions. The reported values represent concentration outputs generated by the manufacturer’s pre-calibrated fluorometric system rather than values derived from an assay-specific calibration curve prepared using serial dilutions of complementary target DNA.

In contrast, the three wild-type samples exhibited significantly lower DNA concentration values (1.7–2.1 ng/µL, mean = 1.9 ± 0.2 ng/µL), while the distilled water control showed a minimal background signal of 2.3 ng/µL. The pronounced difference between the positive and wild-type groups confirms that the CNT–Fe_3_O_4_ nanocomposite can selectively recognize the EGFR T790M mutation with high accuracy, demonstrating negligible non-specific interactions or false-positive signals.^39^, ^40^ The favorable analytical performance observed in the present study can be attributed to the inherent structural and functional advantages of iron oxide-based hybrid nanocomposites. In such systems, the integration of multiple components leads to enhanced surface reactivity, improved structural stability, and tunable physicochemical properties that collectively facilitate efficient interaction with target biomolecules. Recent studies on iron oxide-containing nanocomposites, including CuO@Fe_2_O_3_, α-Fe_2_O_3_@ZnO, and ZnFe_2_O_4_, have demonstrated that these multicomponent nanosystems exhibit improved functional performance owing to their synergistic architecture and optimized surface characteristics. These features contribute to enhanced interaction efficiency and improved signal response in various applications. In agreement with these reports, the CNT–Fe_3_O_4_ hybrid developed in the present work benefits from the combined effects of the conductive CNT framework and the magnetic Fe_3_O_4_ phase, which together provide a stable and functional platform for probe immobilization and effective hybridization. This synergistic behavior likely underlies the clear discrimination observed between mutant and wild-type DNA samples in the fluorometric detection system.^41^, ^42^, ^43^

The enhanced performance of the system can be attributed to two main factors. First, the overnight incubation during the conjugation process likely improved probe surface density and bonding stability, thereby enhancing hybridization efficiency. This observation aligns with previous reports showing that prolonged coupling times significantly strengthen probe–nanoparticle interactions and increase fluorescence response.^44^ Second, the use of EDC/NHS coupling chemistry effectively reduced non-specific adsorption, explaining the consistently low background signals obtained in the wild-type samples and water control.

The fluorometer-derived DNA concentration values obtained from the positive samples were consistently higher than those of the wild-type samples, supporting clear discrimination between the two groups. Comparable studies have shown that nanostructured biosensors exhibit superior sensitivity and specificity relative to conventional PCR-based mutation assays due to their high surface-to-volume ratio and multivalent probe presentation.^45^ The current findings further support these observations, confirming that CNT–Fe_3_O_4_-based hybridization assays can detect clinically relevant mutations even at very low DNA concentrations.

Within the scope of the present proof-of-concept study, the current findings support the feasibility of CNT–Fe_3_O_4_-based hybridization assays for mutation-associated signal discrimination.

Importantly, the consistent concentration values obtained for all T790M-positive samples agree with previously reported nanoparticle-assisted mutation detection strategies designed for liquid biopsy applications^46^, ^47^. These studies collectively demonstrate that nanoparticle-enabled biosensing platforms provide rapid, cost-effective, and portable alternatives to standard PCR diagnostics, facilitating real-time monitoring of resistance-associated mutations in NSCLC patients.^48^

Taken together, the results support the successful functionalization and application of the CNT–Fe_3_O_4_–probe nanocomposite for the selective detection of the EGFR T790M mutation. By clearly distinguishing between multiple positive and negative samples, this study highlights the future potential of the proposed nanoplatform as a promising complementary approach for mutation-associated signal discrimination and its possible adaptation to liquid biopsy applications in precision oncology. The reported concentration values were obtained from three independent T790M-positive samples and three independent wild-type (T790M-negative) samples, with one fluorometric measurement performed for each sample. Therefore, the reported values represent individual sample readouts rather than triplicate technical replicates. The reported mean ± SD values reflect variation among independent samples within each group.

Although established molecular methods are already available for EGFR mutation testing, the present CNT–Fe_3_O_4_–probe platform is not intended to replace routine clinical diagnostics. Rather, this study represents a proof-of-concept investigation designed to assess whether the nanoplatform can selectively recognize the EGFR T790M mutation under controlled experimental conditions. FFPE-derived DNA was used in this initial study because it provided clinically characterized and mutation-confirmed material for preliminary validation. Nevertheless, the greater clinical relevance of this platform may lie in its future adaptation to blood-based testing, particularly in situations where repeat tissue biopsy is risky, not feasible, or insufficient tissue is available. In this context, the proposed system may serve as a complementary mutation-detection approach alongside established molecular assays.

As this work represents a proof-of-concept study performed on a limited number of samples, formal false-positive and false-negative rates were not determined. Further large-scale clinical validation will be required to establish diagnostic performance parameters such as sensitivity, specificity, and predictive error rates.

## 4 Conclusion

In this study, a magnetically responsive and surface-functionalized CNT–Fe_3_O_4_ nanocomposite was successfully developed and employed as a biosensing platform for the detection of the EGFR T790M mutation in NSCLC samples. The nanoplatform demonstrated excellent specificity and sensitivity, as confirmed by the distinct fluorescence differences between positive and negative DNA templates. The reproducible results across multiple samples highlight the robustness and reliability of the nanoparticle–probe conjugation and hybridization processes.

The integration of magnetic separation, EDC/NHS coupling chemistry, and fluorescence-based readout collectively enabled rapid, cost-effective, and precise detection of a clinically significant mutation associated with tyrosine kinase inhibitor resistance. These findings establish a strong proof of concept for utilizing CNT–Fe_3_O_4_–based biosensors in non-invasive liquid biopsy approaches, offering promising potential for early diagnosis, therapeutic monitoring, and personalized cancer management in clinical practice.

## CRediT authorship contribution statement

**Saja S. Falih:** Writing – review & editing, Writing – original draft, Formal analysis, Data curation, Conceptualization. **Ahmed Mahdi Rheima:** Supervision, Methodology, Investigation. **Fatin Fadhel Al-Kazazz:** Supervision, Methodology, Investigation. **Zaid Nsaif Abbas:** Supervision, Methodology, Investigation.

## Declaration of Competing Interest

The authors declare that they have no known competing financial interests or personal relationships that could have appeared to influence the work reported in this paper.
